# Safe-by-Design in Engineering: An Overview and Comparative Analysis of Engineering Disciplines

**DOI:** 10.3390/ijerph18126329

**Published:** 2021-06-11

**Authors:** Pieter van Gelder, Pim Klaassen, Behnam Taebi, Bart Walhout, Ruud van Ommen, Ibo van de Poel, Zoe Robaey, Lotte Asveld, Ruud Balkenende, Frank Hollmann, Erik Jan van Kampen, Nima Khakzad, Robbert Krebbers, Jos de Lange, Wolter Pieters, Karel Terwel, Eelco Visser, Tiny van der Werff, Dick Jung

**Affiliations:** 1 Safety and Security Institute, Delft University of Technology, 2600 GA Delft, The Netherlands; b.taebi@tudelft.nl (B.T.); J.R.vanOmmen@tudelft.nl (R.v.O.); I.R.vandePoel@tudelft.nl (I.v.d.P.); L.Asveld@tudelft.nl (L.A.); A.R.Balkenende@tudelft.nl (R.B.); F.Hollmann@tudelft.nl (F.H.); E.vanKampen@tudelft.nl (E.J.v.K.); r.j.krebbers@tudelft.nl (R.K.); Dutchfarm@gmail.com (J.d.L.); K.C.Terwel@tudelft.nl (K.T.); E.Visser@tudelft.nl (E.V.); 2 Athena Institute, Faculty of Science, Vrije Universiteit, 1081 HV Amsterdam, The Netherlands; p.klaassen@vu.nl; 3 National Institute for Public Health and the Environment, RIVM, 3720 BA Bilthoven, The Netherlands; bart.walhout@rivm.nl; 4 Department of Social Sciences, Wageningen University & Research, 6708 PB Wageningen, The Netherlands; zoe.robaey@wur.nl; 5 School of Occupational and Public Health, Ryerson University, Toronto, ON M5B 2K3, Canada; nima.khakzad@ryerson.ca; 6 Faculty of Social Sciences, Radboud University, 6525 XZ Nijmegen, The Netherlands; wolter.pieters@ru.nl; 7 Directorate Environmental Safety and Risks, Ministry of Infrastructure and Water Management, 2515 XP The Hague, The Netherlands; tiny.vander.werff@minienw.nl (T.v.d.W.); dick.jung@minienw.nl (D.J.)

**Keywords:** safe-by-design, secure-by-design, risk-based design, design for values, responsible research and innovation, uncertainty

## Abstract

In this paper, we provide an overview of how Safe-by-Design is conceived and applied in practice in a large number of engineering disciplines. We discuss the differences, commonalities, and possibilities for mutual learning found in those practices and identify several ways of putting those disciplinary outlooks in perspective. The considered engineering disciplines in the order of historically grown technologies are construction engineering, chemical engineering, aerospace engineering, urban engineering, software engineering, bio-engineering, nano-engineering, and finally cyber space engineering. Each discipline is briefly introduced, the technology at issue is described, the relevant or dominant hazards are examined, the social challenge(s) are observed, and the relevant developments in the field are described. Within each discipline the risk management strategies, the design principles promoting safety or safety awareness, and associated methods or tools are discussed. Possible dilemmas that the designers in the discipline face are highlighted. Each discipline is concluded by discussing the opportunities and bottlenecks in addressing safety. Commonalities and differences between the engineering disciplines are investigated, specifically on the design strategies for which empirical data have been collected. We argue that Safe-by-Design is best considered as a specific elaboration of Responsible Research and Innovation, with an explicit focus on safety in relation to other important values in engineering such as well-being, sustainability, equity, and affordability. Safe-by-Design provides for an intellectual venue where social science and the humanities (SSH) collaborate on technological developments and innovation by helping to proactively incorporate safety considerations into engineering practices, while navigating between the extremes of technological optimism and disproportionate precaution. As such, Safe-by-Design is also a practical tool for policymakers and risk assessors that helps shape governance arrangements for accommodating and incentivizing safety, while fully acknowledging uncertainty.

## 1. Introduction

Under labels such as “mission-oriented research” and “UN Sustainable Development Goals”, funding has been increasing for research and innovation focusing on society’s most pressing challenges, including poverty, climate change, renewable energy, and mobility and health issues [1]. Furthermore, the innovations that assist in dealing with such challenges are not expected to produce new problems and risks themselves. Important research and innovation governance concepts, such as Responsible Research and Innovation (RRI) [2] and Science With and For Society [3], therefore promote the ideas that research and innovation should take place in accordance with societal needs and public values, while also being reflective, anticipative, forward-looking, and responsive [4,5,6].

A key public value that plays a role in technological innovations is safety and a concept in the spirit of RRI dedicated specifically to safety is Safe-by-Design. Although it is still a relatively novel concept, it requires both further elaboration and explicit practical testing. The fundamental idea at the heart of Safe-by-Design is that when innovating, we should try to anticipate risks as much as possible in order to prevent them from happening or to decrease their likelihood because on all relevant measures preventing harm is better than curing its consequences. Thus, we propose that Safe-by-Design can be seen as a heuristic concept that facilitates preventive design practices and builds on the merits of anticipation, inclusion, and responsiveness as highlighted in the literature on the policies stimulating RRI. This conceptualization is a forward-looking method of engaging with issues of risk and safety that requires engineers and innovators to think about the potential risks and hazards the fruit of their work might give rise to throughout its life-cycle. It further facilitates a richer discussion about safety in the broader context of other important public values that engineering design can contribute to, including sustainability, well-being, equity, and affordability.

This conceptualization of Safe-by-Design is the result of a long and still ongoing conversation among this paper’s authors, which include engineers from various disciplines, risk scholars, and scholars of science–society interactions as well as policy-makers and risk regulators in the Netherlands. In this paper, we present an overview of Safe-by-Design as a concept and strategy—or how (realizing) safety has been interpreted and operationalized—in the different fields of engineering. We argue that Safe-by-Design could offer important insights for emphasizing the role of designing for the value of safety from the outset, while also considering other important values in engineering; this approach helps the designer and policy-maker in being more aware of the potential value conflicts and to address them as much as proactively possible. As such, it facilitates well-informed decision-making about risks in engineering and policy. 

Reducing the uncertainties associated with the produced risks was always a major challenge. Different methods have been developed for dealing with this challenge, varying from adding a deterministic safety factor (as an acknowledgement of uncertainties) to using probabilistic approaches based on reducing the probability of certain risks. Probabilistic approaches presuppose that engineer’s possess knowledge with respect to the nature of the risk and that they can calculate its probability of occurrence. They then try to identify potential scenarios, group them into fault and event trees, show how those scenarios could lead to the failure of the system, and eliminate or, as much as possible, reduce the probability of their happening. However, things become more complicated when “knowledge of all failure mechanisms (as well as of further) undesirable consequences that might occur” is not available [7]. This presents a dilemma of control that is more generally represented in the Collingridge dilemma: the further the development of new technology progresses, the more familiar we will be about the technology (and its associated risks) but the less that technology (and associated risks) can be controlled [8]. Since the late 1980s, a new branch has emerged in both scholarly literature and policy documents to consider precaution in innovation or to follow the precautionary principle (PP). This principle has since then been a focal issue in policy discourse in relation to dealing with uncertain risks. 

An important definition of the PP is laid down in The Rio Declaration on Environment and Development (1992); it states that a “lack of full scientific certainty shall not be used as a reason for postponing cost-effective measures to prevent environmental degradation”. The PP literature is full of examples of “late lessons from early warnings”, such as health problems associated with leaded petrol fuel and asbestos-containing construction materials that were not responded to until long after the first problems were observed [9]. The precautionary perspective, also in its more sophisticated interpretations, assumes that the risks of new technologies can largely be anticipated and prevented. In practice, this turns out to be difficult and especially so for new and emerging technologies [10,11]. Risk prevention also involves trade-offs with other values, such as equity, sustainability, and financial costs. Hence, the precaution advocated by the PP always involves discussions about proportionality.

Since the PP is often considered restrictive for innovation, a new development in European policy is promoting the so-called innovation principle (IP). An EU Horizon 2020 call for proposals, for instance, explicitly asked researchers to confront the PP with the IP, “by which potential innovation benefits should be favoured when weighed against potential risks”. Firm believers in the powers of innovation often argue that the risks of possible innovation can best be addressed by technological means and that researchers do not have the option of not innovating at all because of potential risks. This paper addresses the question of how Safe-by-Design can strike a balance in enabling innovation as much as possible, while ensuring that innovations do not carry risks and impose undesired impacts. 

A key question in this respect is how to link such a rather general normative starting point to the practical challenges different engineering disciplines are concerned with. In this paper we will provide an overview of different factually performed operationalizations of Safe-by-Design. In displaying the state of the art applications of Safe-by-Design in different engineering disciplines, its strengths, weaknesses, and blind spots come to the surface. Building on our analysis here, we propose a method going forward in using the concept of Safe-by-Design for aligning innovation and precaution. Two remarks are in order here. First, the notion of Safe-by-Design has sometimes been referred to as Safety-by-Design [12] or Design for Safety, depending on the academic fields [13]. We consider these differences to be mostly semantic; the basic rationale of all these approaches is pro-actively assessing and addressing safety issues. In this paper we will consistently use Safe-by-Design. Second, while the primary focus of our conceptualization is safety, in different instances we will implicitly discuss security issues as well. Safety in our approach is the result of an accident while security has a component of intentionality. In Table 1, for instance, where different design strategies for designing safety have been discussed, we also included “vandal proof design” or designing against (intentional) vandalism. While tangentially included in parts of our approaches and discussions, security is an important key value that needs to be explicitly addressed. Returning to the same example, while “vandal proof design” helps to protect against vandalism, engineering designs could also be target of terrorist attacks or attacks of adversary states. 

The paper is organized as follows. We first provide a brief background on some of the key concepts in this article (Section 2) followed by a description of our research approach (Section 3). We then provide an overview of how Safe-by-Design is conceived and put into practice in different engineering disciplines (Section 4). In Section 5, our diverse findings from different engineering disciplines converge. First, we discuss the differences and commonalities and list a numbers of design strategies we have come across in different engineering perspectives (Table 1). We then investigate the manifestations of different strategies in different disciplines (Table 2). Finally, in Section 6, we argue that Safe-by-Design is best considered as a specific elaboration of Responsible Research and Innovation, with an explicit focus on safety in relation to other important values in engineering design. Safe-by-Design, then, could provide an intellectual venue where social science and humanities (SSH) collaborate on technological developments and innovation by helping to proactively incorporate safety considerations into engineering practices while navigating between the extremes of technological optimism and disproportionate precaution.

## 2. Basic Concepts 

This article’s main structure builds on a specific understanding of several basic concepts—engineering, safety and security, and addressing safety and security—which we will briefly introduce here.

*Engineering*: Engineering is concerned with the creation of systems, devices, and processes. Engineering disciplines and professions apply scientific theories, mathematical models, and empirical evidence to design, create, and analyze technological solutions useful to and sought by society. Engineers therefore need to be familiar with not only natural laws but also safety risks, juridical laws, and regulations as well as a wide variety of (human) factors pertaining to needs, values, perception, acceptance, usability, and costs. Engineering is conventionally subdivided into the branches of civil, chemical, electrical, and mechanical engineering [14]. The Accreditation Board for Engineering and Technology (ABET) distinguishes between engineering design and engineering science. They define engineering design as a process of devising a system, component, or process to meet desired needs and specifications within constraints [15,16,17]. It is an iterative, creative, decision-making process in which the basic sciences, mathematics, and engineering sciences are applied to convert resources into solutions. Engineering design involves identifying opportunities, developing requirements, performing analysis and synthesis, generating multiple solutions, evaluating solutions against requirements, considering risks, and making trade-offs for the purpose of obtaining a high-quality solutions under the given circumstances. Engineering sciences are based on mathematics and basic sciences but carry knowledge further toward creative application needed to solve engineering problems. These studies provide a bridge between mathematics and basic sciences on the one hand and engineering practices on the other.

*Safety and security*: Safety is an important value in any engineering design. It is the state of being “safe”, the condition of being protected from harm, or other non-desirable outcomes. Safety can also refer to the control of recognized hazards to achieve an acceptable level of risk. These can be hazards of any type, including natural hazards, technological hazards, exposure hazards due to toxic emissions, and hazards caused by human action or inaction. Safety is distinct from security in that the former relates to unintentional and random factors, while the latter is related to intentional and malicious factors.

*Addressing safety and security*: Safety and security can be addressed at all phases of the life cycle of any product, process, or system (i.e., plan, conceptual design, detailed design, optimized design, test, implement/build, operate, maintain, dispose, or reuse). By definition, Safe-by-Design concentrates on the plan and design phases. It aims at including safety as a value to be translated into design requirements from the earliest stages of product and process development onwards. This implies addressing questions such as the following: What could go wrong with this design in its intended or unintended use? Which components and structures are potentially dangerous? How can the design be adapted to prevent the occurrence of risks, for instance, by replacing, changing, or reducing components? If things do go wrong, how can adverse effects be prevented or controlled? 

We approached this exploration of the significations given to Safe-by-Design with these understandings in mind. The following section describes our approach.

## 3. Research Approach

The commonalities and differences in the conceptions of Safe-by-Design as well as the possibly transferable lessons from our analysis are based on an inventory of meanings ascribed to hazard, safety, and Safe-by-Design in eight engineering faculties at the Delft University of Technology. These faculties are (1) Technology, Policy and Management (TPM), (2) Industrial Design Engineering (IDE), (3) Civil Engineering and Geosciences (CEG), (4) Architecture and the Built Environment (A&BE), (5) Applied Sciences (AS), (6) Aerospace Engineering (AE), (7) Mechanical, Maritime and Materials Engineering (3mE), and (8) Electrical Engineering, Mathematics and Computer Science (EEMCS). Representatives from each of these faculties contributed to this inventory by providing the individual disciplinary descriptions found in Section 4. Each researcher answered the same set of questions, which was collectively developed during a series of workshops and meetings held between June 2018 and May 2020. During these meetings, the concept of Safe-by-Design, the promise it holds, and the questions it does not (easily) answer were repeatedly discussed with policymakers from the Dutch Ministry of Infrastructure and Water Management and researchers from the Dutch National Institute for Public Health and the Environment (RIVM). 

Box 1 displays the elements used to collect pertinent information about each discipline involved in this study. These elements provided the structure for bottom-up descriptions of what meaning the disciplines give to various concepts involved in understanding how Safe-by-Design is or could be operationalized and to explore to what degree Safe-by-Design is already implemented. Our aim was to understand what safety risks are present in different disciplines and what addressing risks early on in research and innovation requires without steering those accounts too much on the basis of a preconceived and elaborate conceptualization of Safe-by-Design. Bringing together these accounts enables an in-depth understanding of the relevant commonalities and differences between these disciplines and of the potential characteristics different disciplines possess that could help increase understandings and possible meaning(s) of Safe-by-Design. The experts who contributed their disciplinary perspectives are all (associate) professors in their fields and co-authors of this article and they all work or have worked at Delft University of Technology.

Box 1 **Analytical template for collecting disciplinary conceptions of Safe-by-Design.**

**Context** Brief introduction to the disciplinary domain, hazards, social challenge(s), and relevant developments in the field. **Focus** What is the “system” that the discipline’s design practice focuses on? What are the technological and/or social components? Which risk management strategies and associated methods or tools are used? Are there (codified) design principles promoting safety or safety awareness and, if so, what are they? What considerations or dilemmas do designers in the discipline face? **Outlook** What does the future of the discipline and/or the system affected by its research look like? Where are the opportunities and bottlenecks in addressing safety?

## 4. Disciplinary Perspectives

The upshot of the research approach that was described above is that the next eight subsections present a wide variety of methods for thinking about safety risks. Individual co-authors were given and have taken quite some liberty in elaborating what they perceive to be the relevant aspects of context of their disciplinary domain for furthering our conceptualization of Safe-by-Design and the same holds for their conceptions of the system their work focuses on and the future outlook of their discipline. Thus, this section shows the state of the art methods in current practices of dealing with safety risks in what are quite divergent areas of study. Insofar as the conceptualization of Safe-by-Design put forth here does not resonate with the perspectives presented here, it is because there still exists room for improvement.

As a final preparatory remark, let us point out that the disciplinary grouping used here is rather crude. Although this certainly impacts what safety risks are identified as well as the descriptions of how they are to be dealt with, we believe that at the present stage of conceptualizing Safe-by-Design a finer and more detailed grouping would unnecessarily complicate the discussion.

### 4.1. Construction Engineering

#### 4.1.1. Context: Human Factors in Distributed Settings

The building industry is responsible for the design, engineering, and construction of buildings, infrastructure, and other engineering structures, such as pipelines and wind turbines. In The Netherlands, this industry accounts for approximately 10% of GDP within the country.

Failures of these structures occur worldwide. The individual risk of death related to structural failure is very low, but it is estimated that failures add approximately 10% to the construction costs of structures.

A failure occurs when the loads acting on a structure (such as wind load, seismic load, live load, etc.) exceed the strength of the structure. Investigations have shown that failures occur relatively often in foundations, floors, and facades and less often in columns. Failures can often be traced back to the structural design before implementation. Furthermore, failures also occur during the construction phases and use phases. In a number of cases, the causes of failure are force majeure, but the human factor does count significantly.

Several studies have concluded that design and construction errors during the construction process were the main causes of collapses and minor failures. Terwel and Janssen [18] reported that the influencing factors for structural safety within the design and construction process are mainly those that are related to organizational factors, such as interrelationships between different project partners, particularly the lack of communication and cooperation, lack of control mechanisms, lack of assignment of responsibilities, lack of structural risk management, lack of safety culture, and lack of knowledge infrastructure. 

#### 4.1.2. Focus: Structural and Organizational Measures

Structural engineering is the design and engineering part of the building industry. Errors in these areas are estimated to account for approximately 50% of structural failures. The structural engineering system is complex and can be decomposed into different levels on which the design practice focuses on the following:

- Macro level: external factors such as legislation, climate, politics, and culture;
- Meso level: business and project factors such as safety culture and working; conditions
- Micro level: human factors such as competence, stress resistance, knowledge, and attitude.

Safety calculations in the European construction sector are based on load and material factors and follow the Eurocodes. Although human factors in design and user errors cause about 90% of the failures, they are not included in the calculations. Therefore, additional structural and non-structural measures are needed. Structural measures that can increase structural safety include using ductile materials rather than brittle materials and over-designing the structure (e.g., by adding redundant elements). A robust or resilient structure is a structure that will not fully collapse after local damage has occurred. Non-structural measures can also increase structural safety. Examples include reducing errors in the design phase by appointing an integral design officer or capable coordinating structural engineer, delineating responsibilities, and establishing extra supervisory control actions. Engineers can also be certified, which can guarantee a minimum level of competency.

The following nine rules can further increase the level of structural safety in future construction projects:

- Keep the construction project and process simple;
- Provide sufficient resources and suitable people to fit the complexity of the project;
- Create a complete list of tasks and responsibilities and check and act on each;
- Provide a competent chief constructor with a certain level of accountability and responsibility;
- Pursue repetitive forms of collaboration;
- Develop safety awareness;
- Ensure effective exchange of information and knowledge;
- Implement effective risk management of the process;
- Support (inter)national initiatives in the field of structural safety and include them contractually where possible.

Designers face a strong dilemma between costs and safety in the choices they have to make. A construction project involves many different actors and parties and often many small contractors are working on an assignment for the lowest possible price. In addition, the culture within the construction sector is anti-authoritarian. Engineers’ financial liability is low and no higher than the total amount of the contract. Safety measures are often considered as additional costs and are therefore usually reactive in their application; as soon as something goes wrong, measures are taken.

#### 4.1.3. Outlook: Digitalization and Automation 

One challenge for the industry is increasing the use of RFID technology (radio frequency identification), BIM (building information modelling), and computers, all of which offer opportunities for complex design and increases engineering speed. These technologies may bring benefits [19] by improving real-time information visibility and traceability to the management of people, materials, and machinery for construction projects. However, the construction industry has been slow to adopt these technologies mainly because of the many technical, financial, and ethical hurdles involved. These technologies also require a thorough understanding and checking of designs. 

### 4.2. Chemical Engineering

#### 4.2.1. Context: Large-Scale Industry Response to Accidents and Pollution

The chemical industry is concerned with the processing of bulk resources into other products. Process industry accidents can result in the loss of both properties and lives [20]. Examples of major accidents are “Seveso”, “Bhopal”, and “Sandoz”. Taylor [21] showed that the causes of process industry accidents are related to chemical properties, operation issues, human errors, or inadequate process design. 

Today’s chemical industry is actively investing in safer production systems and in the design of safer products. These efforts are flanked by ever more restrictive legislation (such as REACH). Major chemical producers such as BASF, DSM, Dow, and Evonik as well as suppliers are heavily investing in responsible manufacturing. 

Safe-by-Design in the process industry focuses on preventing leaks, spills, fires, explosions, equipment malfunction, over-pressures, over-temperatures, corrosion, metal fatigue, and similar conditions in chemical process facilities dealing with hazardous materials, such as refineries and oil and gas (onshore and offshore) production installations.

#### 4.2.2. Focus: Consolidated Principles for Safe and Green Chemistry

Process design is about the choice and sequencing of bulk resources for the desired physical and chemical transformation of materials. The design involves process flow diagrams, which usually include a material and energy balance showing typical or design flowrates, stream compositions, and stream and equipment pressures and temperatures. The design also involves piping and instrumentation diagrams that show each and every pipeline, with piping class, pipe diameter, and valving along with instrument locations and process control schemes.

In addition to the technical components, the process industrial system also includes human and organizational factors that influence the system’s performance. Performance-shaping factors affecting human performance at different levels of task complexity and in multiple categories of safety culture are applied at descriptive, observational, and prescriptive levels in the process industry. 

Kletz [22] introduced seven qualitative principles for inherently safer design:

1. Minimize: Reducing the amount of hazardous material present at any one time (e.g., by using smaller batches);
2. Substitute: Replacing one material with a less hazardous one (e.g., cleaning with water and detergent rather than a flammable solvent);
3. Moderate: Reducing the strength of an effect (e.g., having a cold liquid instead of a gas at high pressure or using material in a dilute rather than concentrated form);
4. Simplify: Eliminating problems by design rather than by adding equipment or features to deal with them. Fitting options and using complex procedures only if they are really necessary;
5. Improve fault tolerance: Designing equipment and processes to be capable of withstanding possible faults or deviations from design.
6. Limit effects: Adjusting design, location, or transportation of equipment so that the worst possible condition produces less danger (e.g., having gravity take leaks to safe places; using bunds; avoiding knock-on effects);
7. Make fool proof: Making incorrect assembly impossible; ease of control.

While the above seven points are mainly concerned with the operation (preventing accidents), Anastas and Warner [23] extended these by adding sustainability related points, resulting in the following 12 principles: (1) Prevention instead of remediation, (2) Atom economy as a guiding principle for synthesis planning, (3) Less hazardous chemical syntheses, (4) Designing safer chemicals, (5) Safer solvents and auxiliaries, (6) Design for energy efficiency, (7) Use of renewable feedstocks, (8) Reduce derivatives, (9) Catalysis, (10) Design for degradation, (11) Real-time analysis for pollution prevention, and (12) Inherently safer chemistry for accident prevention. These principles can serve as a guideline in various aspects:

- Less hazardous chemical syntheses implies a radical rethinking in the design of chemical synthesis routes. New catalytic routes reducing the number of synthesis steps are one major pillar here. Other changes include replacing problematic (reactive or environmentally questionable) solvents and reagents with safer options and using catalysis to lower reaction temperatures and thereby reduce explosion risks. New concepts such as cascade reactions (reducing the number of individual synthesis steps including downstream processing) or reactor concepts such as flow chemistry are also gaining interest.
- Designing safer chemicals aimed at higher-quality products (i.e., lower amounts or absence of unidentified and potentially toxic by-products). Catalysis, in particular, is a key element in achieving this goal. Furthermore, using selective catalysts (especially biocatalysts) improves the selectivity of the reaction, thus yielding fewer or no undesired side-products. This abolishes or drastically reduces the need for derivatization steps and the need to remove auxiliaries from the final product.
- Production of products has to be sustainable to “satisfy today’s needs without compromising the resources of the following generations” [24].
- New energy-saving syntheses will conserve fossil resources; the same is true for using non-noble metal catalysts and auxiliaries (e.g., avoiding non-renewable phosphates or helium).
- There is also an ongoing trend towards a more holistic definition of product performance. While, traditionally, this term has been applied to the designed use of a given product, it is now being extended to earlier and later phases of the product’s life. For example, new feedstocks are being explored to substitute fossil-based polymers with renewable alternatives. Equally important are the current efforts to design polymers with built-in predetermined breaking points to facilitate their recycling and their natural degradation if exposed to the environment (avoiding massive accumulation of wastes in the oceans, for example).
- Process designers aim to maximize throughput rate, process yield, and product purity, while simultaneously limiting capital, operation, and maintenance costs; space required; safety concerns; environmental impacts; and emissions and waste production. They also have to consider the minimum levels of reliability, redundancy, flexibility, and anticipated variability in feedstock and product. Several hazard indices have been developed as tools for chemical process loss prevention and risk management. Each provides a dimensionless index value that is defined relatively and may be combined with a decision analysis tool for setting priorities.

#### 4.2.3. Outlook: From Safety and Sustainability to Non-Toxic and Circular Economy

The future of chemical engineering is heading towards a circular economy in which “waste” as a concept will disappear. Wastes will be perceived as feedstocks for new products. Therefore, the design of tomorrow’s chemicals and materials should take sustainability into account. Chemicals and materials and their production processes must be:

1. Based on non-depleting resources: that is, transitioning from fossil-based chemicals to renewable feedstock. Moreover, anthropogenic CO_2_ will be used as feedstock.
2. Non-toxic: necessitating more predictive models for structure-activity relationships.
3. Non-persistent: built-in (bio)degradability of products that are ultimately distributed into the environment (e.g., consumer products such as cosmetics and active pharmaceutical ingredients).

In the chemical engineering domain, the above design principles are frequently used by academic and industrial researchers as a “tick list” to prove safety and “greenness”. However, a holistic and quantitative evaluation and comparison with existing alternatives is necessary to be able to claim environmental, safety, or societal benefits. 

Today, some of the principles have been standardized in “life cycle analysis” approaches (e.g., ISO 14040:2006), but these require extensive data and are consequently too laborious and costly for researchers. Simpler semi-quantitative methods such as the “E-factor” are available and should be used more frequently [25].

Furthermore, several measures of inherent danger have been developed and are in further development by researchers such as Gentile et al. [26], Khan and Amyotte [27], and Tugnoli et al. [28]. One of these measures is the DOW fire and explosion index (F&EI), which relies mainly on the material factor, consisting of the flammability and reactivity of chemical substances. The DOW F&EI assesses the hazardousness of a process unit (e.g., a storage tank) merely on the basis of the type and inventory of the contained chemical without considering the process unit’s impact on adjacent units via potential domino effects.

Reliable metrics based on graph theory (e.g., out-closeness and betweenness) have therefore been developed to assess the criticality of process units with regard to domino effects [29]. The integration of graph metrics with the DOW F&EI is expected to reflect a more realistic and accurate measure of the hazardousness of a process unit in chemical/process units, which in turn can be considered during the fail-safe/fail-secure designing of chemical plants or in the optimal allocation of safety/security measures.

Finally, Safe-by-Design and Sustainable-by-Design should become fully integrated in education.

### 4.3. Aerospace Engineering

#### 4.3.1. Context: Integrated Sector and Safety Culture

Commercial air transportation is one of the safest modes of transportation [30]. Although flying is relatively safe, there are still accidents, sometimes with many casualties, which intensifies the effect these incidents have on society and on the perception of air transportation safety. This has created a very strong safety culture within aviation, with many regulatory bodies overseeing the design and operation of aircraft as well as training and maintenance. The commercial air transportation sector is characterized by a widely distributed network of component suppliers and operations (airlines) but a limited number of final assembly manufacturers.

#### 4.3.2. Focus: Flight Control Systems as Part of a Layered Safety Approach 

Safe-by-Design for aircraft is a multi-layered approach that includes diverse topics, such as material selection, structures, stability and control, fault detection and isolation, human-machine interface design, pilot training, air traffic control, maintenance, and certification. Due to the variety of these different aspects, we will focus on the layered safety approach in general and on flight control system design in particular.

Safety in aircraft design is mainly based on the redundancy of critical systems. There are double, triple, and sometimes quadruple redundant systems for critical components related to flight control (sensors, flight control computers, and control surfaces such as elevators, flaps, or ailerons) as well as multiple redundant modes in the software systems, for example, the multiple flight control laws in Airbus aircraft [31].

In addition to the redundancy in subsystems, the airframe itself is designed in such a manner that it is naturally stable in flight. Thus, even without any control surface or engine inputs from either the human pilot or the automatic pilot, the aircraft will glide in a stable manner and rejects disturbances, such as those from gusts.

Airworthiness authorities also play a crucial part in the safety of aviation. They ensure that the aircraft satisfies the airworthiness criteria and they oversee the whole “chain” from design and manufacturing to operations and maintenance, including pilot licensing and air traffic control. Many safety-related design criteria, such as those related to handling qualities (characteristics of a flight vehicle that govern the ease and precision with which a pilot is able to perform a flying task), are specified in airworthiness regulations [32]. Aircraft manufacturers must demonstrate that their aircrafts satisfy these criteria in a certification process.

An important choice in aircraft design is the role of automation in protecting the safe flight envelope. Airbus traditionally follows a stronger automation principle in which pilot input is closely monitored and checked, which makes it impossible for the pilot to provide inputs to the aircraft that would be considered too dangerous. In contrast, Boeing’s philosophy has always been more focused on manual control and gives pilots more freedom to control the aircraft but still warning them if they are approaching the edge of the safe flight envelope. This design choice is related to the trust engineers have in automation. We see accidents partly caused by humans that could have easily been avoided by automation. However, accidents have also been caused primarily by automation, where the human is so far removed from the primary operation of the system that, even if automation is switched off, the reduced awareness of the (upset) situation still leads to accidents. 

#### 4.3.3. Outlook: Safe Automation

Recent years have shown an increase in the autonomy of aerial vehicles, through the increased usage of unmanned aerial vehicles and the development of intelligent adaptive flight control systems for manned aviation, such as personal air vehicles. The challenge in designing autonomous aerial vehicles is coping with situations (e.g., failures or disturbances) during the operational phase that were not expected during the design phase. Under manual control of these vehicles, human pilots can adapt their strategies to cope with these situations. In classical automatic flight control system design, the control system is only designed to cope with specific known situations and it cannot adapt its strategy autonomously.

Reinforcement learning (RL) is a framework of machine learning techniques, based on human-like learning from experience, that can be used to design adaptive control systems for autonomous vehicles. There have been some initial applications of RL in automatic flight control system design [33,34], but the main challenge is to guarantee safety of learning. Since RL is essentially learning by trial and error, mistakes have to be made before something can be learned from those mistakes. However, the mistakes should not be so big that the learning cannot continue. In the literature, this challenge is also referred to as safety of exploration [35].

There is a trade-off between the adaptiveness of the control system, which increases aircraft safety in unanticipated situations and the inherent risks of learning from experiences, which can decrease safety. It is up to the designer of future intelligent flight control systems to balance both aspects and to add constraints to the adaptiveness or authority of the adaptive system in order to ensure safety. Airworthiness authorities also play an important role here because current regulations are not designed for adaptive systems. This means further development on their side is required before these adaptive systems can be certified.

### 4.4. Urban Environment

#### 4.4.1. Context: Crime Prevention as a Distinct Aspect in Urban Design

Secure-by-design in the urban built environment concerns the form, arrangement, and design of buildings and public spaces that can encourage or discourage criminal activity and undesirable behavior. The design methodology is also referred to as “crime prevention through environmental design” (CPTED) as an agenda for adjusting the built environment to create safer neighborhoods. It originated in the US around 1970 [36], when urban renewal strategies were perceived to be destroying the social framework required for self-policing. It is based on principles from architecture (with the concept of “defensible space”) and criminology. In their meta-analysis of multiple-component CPTED initiatives, Casteel and Peek-Asa [37] found that robberies decreased by 30–84% in US neighborhoods with CPTED initiatives compared to those without such initiatives. 

The design practice focuses on the built urban environment and involves architects, urban space planners, law enforcement professionals, and criminologists among others. It focuses on physical changes made to the built environment, but from a technological and social point of view, such that it changes the human perception of risk, for instance by planting trees and shrubs, using lighting correctly, and encouraging pedestrian and bicycle traffic in the streets.

#### 4.4.2. Focus: Inhibiting Crime

Three (non-codified) design principles can increase safety and security levels in the urban environment:

1. Natural Surveillance: People are less likely to be violent or take part in illegal activities if they know they can be seen. This can be achieved by keeping areas well lit, increasing presence in high traffic areas, and eliminating hiding places.
2. Territorial reinforcement and access control: This can be achieved by clearly defining the boundaries between public and private areas with fencing, landscaping, and signs. Well-marked areas direct the flow of traffic and discourage non-local traffic from passing on private grounds.
3. Maintenance: This refers to keeping buildings properly maintained by quickly removing graffiti and trash, fixing broken windows, keeping school hallways clear, and cleaning landscaping. The idea is that “signs of disorder” attract disorderly behaviour that may turn into violent acts.

Secure-by-design in the built environment requires typical measures such as additional space, more lightning, and so on, which have a direct influence on the costs of building such urban environments. Modifying an existing environment to comply with the CPTED principles can be costly, but when included in the original design phase of the built environment, the cost of secure-by-design can be reduced. By incorporating the potential cost reduction in crime prevention, CPTED principles become quite attractive and cost-effective.

#### 4.4.3. Outlook: Limits to Security?

Opportunities exist to further increase the effectiveness of secure-by-design principles in the urban environment and to derive additional best practices. However, they face constraints regarding the question of how much crime prevention is really required for a particular place. How much freedom should a community give up, usually expressed in terms of freedom of movement and gathering options, to be free from the fear of crime? Some stakeholders have suggested that a risk management approach may be better than an approach driven by fear. At the same time, there is a worldwide increase in gated communities or protected communities and in the use of camera systems in public spaces.

### 4.5. Software Engineering 

#### 4.5.1. Context: Safety as Performance Requirement

More and more processes in society rely on software. It is therefore critical to ensure that such software is safe: that it does not crash, is secure, performs at expected levels, and so on. Unfortunately, software development is error prone and errors often end up in software used in production. Consequently, a lot of money is spent on repairing and maintaining erroneous software and errors that remain undetected can have disastrous consequences (e.g., Heartbleed [38] and Toyota [39]). The Safe-by-Design principle promotes the importance developing techniques and tools that prevent software errors early in the software development process.

Depending on the application domain, software needs to satisfy a combination of different properties to guarantee its safety. For example:

- The software is free of anomalies that may cause it to stop functioning or to have erroneous behaviour (e.g., race conditions, dead locks, or buffer overflows).
- The software functions according to a specification of its behaviour (i.e., given input satisfying P, the software’s output satisfies Q).
- The software satisfies certain performance requirements such as worst-case execution time (WCET) or memory usage requirements.
- The software has security properties such as integrity, confidentiality, or availability.

Violation of these properties may lead to economic loss, loss of privacy, loss of human lives, or other accidents. Therefore, an appropriate set of measures is required to obtain an adequate level of trust in these properties.

#### 4.5.2. Focus: Trade-Offs and Choices in Safety Approaches 

Software developers use a combination of approaches to ensure software safety:

- Dynamic analysis and testing: In this approach, software is run on a real platform (testing) or an instrumented platform (dynamic analysis) with a representative set of inputs to confirm if it satisfies the desired properties. This approach can be used at the level of individual software modules (unit testing), against interfaces between modules (integration testing), at the level of the whole software (system testing), or at the level of the interaction between the software and the physical system (acceptance testing). Since only a finite number of inputs can be tested, testing or dynamic analysis can never guarantee the absence of software errors. It is therefore crucial to write representative tests that lead to good coverage of the different modules of the software.
- Static analysis and formal verification: This approach aims to establish properties at the level of the source code without actually running the software. Contrary to testing, static analysis or formal verification can ensure that properties hold for any input. However, this comes with a trade-off. This approach can typically establish either fairly weak properties, such as the absence of anomalies (through static analysis methods such as abstract interpretation or type systems) in a fully automatic manner; or strong properties such as correct input/output behaviour (through formal verification methods such as model checking, deductive verification, or theorem proving) with significant human guidance.
- Design patterns and coding conventions: This approach aims to write software in a structured method by using reusable patterns for common problems (design patterns) and by following certain conventions for organization (coding conventions). This approach typically goes hand in hand with testing in early phases of development (test-driven development) and with using static analyses to enforce that certain patterns are being consistently used (e.g., through linter tools).

Applying these approaches comes with a number of choices. The first dilemma is what properties the software should have. Some properties (e.g., the absence of anomalies) are independent of the application domain and are easy to specify, while other properties (e.g., properties about the behavior of the program) are dependent on the application domain and are very hard to specify.

The second dilemma is what approach to use to verify these properties. There are a number of trade-offs involved: the amount of time/money required, what kind of properties are guaranteed to hold, whether the properties are guaranteed to hold probabilistically or for any input, whether the properties hold for the real system or a model of the system, how much human guidance is needed, and so on.

For application areas where safety is essential, a combination of safety approaches should be used to establish a combination of properties. For ordinary software, there is little consensus on what these combinations should be, but for safety critical software various standards specify what approaches should be used to achieve a certain level of trust: IEC-15408 (security), IEC-61508 (electronic systems), DO-178B/C (airborne systems), and ISO-26262 (road vehicles).

#### 4.5.3. Outlook: Software Solutions for Software Safety

Ideally, software developers should create the means to develop software that is guaranteed to have no errors. However, this is not possible. First, it would require careful specifications of what it means for software to have “no errors”, which is a hard problem and requires anticipation of everything that could go wrong. Second, even with such specifications at hand, Rice’s theorem [40] establishes that it is impossible to automatically establish that software has non-trivial specifications. That is, software developers cannot write a program that checks that another program has all the properties they would like it to have. As a consequence, ruling out errors in software will always involve work by people.

An important direction for future research is to develop stronger methods for static analysis and formal verification that can establish stronger properties with less human guidance. Over the last few years, much success has been gained in this direction. For example, developers have been able to statically verify strong specifications of core software such as compilers (CompCert) and operating systems (L4verified).

Moreover, an alternative to relying on programming discipline could be improving the software development environment so that violations of safety properties are discovered during development, thereby making the software Safe-by-Design. There are different complementary routes to programming environments that support the design of safe software. One example is to develop programming languages that are more appropriate for specific domains.

### 4.6. Biotechnology

#### 4.6.1. Context: Heavily Regulated Platform Technology

Biotechnology encompasses a range of techniques that use biological knowledge to provide functional gains to living organisms. These gains-of-function fulfill specific goals in industrial, medical, and agricultural biotechnology. In each of these fields, biotechnology can contribute to improving human lives through cleaner and sustainable production processes, through plants or animals that can better deal with production challenges like diseases or pests, through microorganisms that are capable of dealing with wastes and recovering important nutrients, or through innovative diagnostic methods and drug delivery.

Biotechnology is an enabling technology with such a wide range of applications that discussions of risks and safety must always be tied to specific areas of application. Risk management strategies are likely to differ from case to case and certainly from sector to sector. Additionally, there can potentially be human, social, economic, or environmental risks. In this overview, only risks to the environment and humans are considered for analysis because the focus here is on current practices in and challenges for Safe-by-Design and because these are the risks bioengineers currently design for. Addressing other types of risks requires other methods and design considerations, which would go beyond the scope of this overview.

#### 4.6.2. Focus: Safe-by-Design Principles

Safe-by-Design is not yet a widely recognized concept in the biotechnology community. However, since 2009, Safe-by-Design has been gaining some traction in the synthetic biology community [41], where designing organisms is considered through the assembly of BioBricks^TM^ or building blocks of life in model organisms. Thus, Safe-by-Design rationales in biotechnology mainly concern microorganisms and only address the following potential hazards [42]:

- Toxicity and pathogenicity to humans, other animals, and plants;
- Persistence and invasiveness in ecosystems;
- Horizontal gene transfer and gene pool contamination in populations.

The interest in Safe-by-Design also stems from the potential it offers to safely and deliberately release modified microorganisms into the environment, which could contribute to solving global challenges, such as microbial antibiotic resistance [43]. Current Safe-by-Design strategies in biotechnology (adapted from Robaey [42]) are as follows:

- Choosing the right organism: to minimize toxicity, pathogenicity, and potential invasiveness, provided the designer takes context into account;
- Designing physical barriers: to create barriers at different scales;
- Self-destruct mechanisms: to trigger events leading to cell death if certain external conditions change (e.g., kill-switches);
- Dependency: to require certain elements or food to survive (e.g., auxotrophy);
- Design distance between the natural and the synthetic: to minimize exchange of genes between organisms (e.g., orthogonality, xenobiology, and recoding the genome);
- Sculpting evolution: to influence the genetic make-up of a population (e.g., daisy drives);
- Control with external stimuli: to activate or deactivate cells by using external stimuli (e.g., light);
- Warning mechanisms: to require human intervention, with the aid of sensors in the microorganism (e.g., visible change in colour to signal changing conditions that can affect safety).

#### 4.6.3. Outlook: Broadening the Scope from Control to Choice

Choosing what strategies to implement is not a clear-cut process for bioengineers. Some strategies, such as choosing the right organism, are established elements of current safety practice that are even seen as common sense. Most other strategies, however, are still in the research stage. These strategies are driven by scientific curiosity more than by application needs [42,44]. Indeed, implementation of Safe-by-Design strategies in biotechnology faces several challenges at both technical and societal levels.

From a technical perspective, a variety of questions emerge: First, how safe is safe enough? How many of these strategies are needed to guarantee an acceptable safety level? Who gets to decide on this level of safety? Second, what does Safe-by-Design mean for complex organisms? Third, considering the various domains biotechnology features in, which risks can Safe-By-Design actually address? Last but not least, for living technologies subject to evolutionary forces, what are the other methods of thinking about safety that do not rely only on the idea of control [45]? 

These technical questions require societal deliberation, but they are also accompanied by another set of societal questions: what are other safety strategies that could be part of a Safe-by-Design concept not necessarily by designing safe microorganisms but by designing for safety or by organizing responsibility for safety [7]? Considering Safe-by-Design strategies for microorganisms in containment or when released into the environment amounts to considering different sociotechnical systems in the design practice. For instance, different stakeholders will perceive different risks and it is unknown whether and how all of these can be addressed [46,47]. Moreover, such considerations reintroduce the question of how safe is safe enough.

Safe-by-Design offers opportunities for discussions on safety, on early choices in the design of organisms, and on involving different stakeholders in the process. Formulating design principles for safety in biotechnology will require the integration of both technical and societal challenges.

### 4.7. Nanomaterials 

#### 4.7.1. Context: Emerging Technology, Emerging Risks 

In recent years, chemistry has focused on increasingly larger molecular and supramolecular assemblies, while engineering has been able to make increasingly smaller devices. These developments intersect in the field of nanotechnology. Important steps forward have been made, yielding novel nanomaterials such as graphene, carbon nanotubes, and quantum dots [48]. Nanosized objects often possess very different properties than their bulk counterparts: for example, gold nanoparticles have a much lower melting point than a block of gold [49].

Smart materials based on nanotechnology have the potential to enable breakthroughs in seemingly unrelated fields from personalized medicine without side-effects to high-power fast-charging batteries. These materials can play an important role in addressing societal challenges. However, insight into their effects on human health and the environment is still limited [50]. While for some nanomaterials the kind of risk they pose has become clearer for some, for others it is still unknown or difficult to determine.

This overview focuses on the design of nanostructured materials for safe usage, keeping in mind that the required Safe-by-Design strategy will depend on the specific application. In practice, different names are used: for example, nanostructured materials, engineered nanomaterials, or, briefly, nanomaterials. These materials contain building blocks with one or more dimensions below 100 nm: e.g., nanoparticles, nanocylinders, or nanosheets. While the focus here is more on the design aspects, other efforts such as the EU projects NANoREG and NanoReg2 have been focusing more on screening and assessing safety levels [51,52].

#### 4.7.2. Focus: Design Options

Strategies for guaranteeing safety when applying nanomaterials are still under development. Morose [53] proposed five design principles for safer designs involving nanomaterials: size, surface, and structure; alternative materials; functionalization; encapsulation; and reducing quantity. This set of principles is not broadly accepted, but several components of it are supported by other authors. A review of recently published work (e.g., [54,55,56]) resulted in the following slightly adapted set of six design aspects that should be considered: materials, morphology, clustering, coating, embedding, and minimizing quantity.

1. Material choice is crucial. Certain ceramics, such as silica and alumina, are largely inert and therefore much safer to use than several metals. However, this is not a guarantee: the very small size and/or specific shape can lead to the reactivity of materials that are inert at the macroscale. For example, aluminum at larger scales is inert, but aluminum nanoparticles can serve as rocket fuel [57]. Carbon is normally safe for human health, but carbon nanotubes pose severe health risks [58,59].
2. Morphology covers several important properties, including size and shape. Although all particles smaller than 100 nm are considered nanoparticles, many of the special functionalities only start when the particles are much (~10 times) smaller. Several researchers suggest that particles < 30 nm are much more toxic to the body than particles in the 30–100 nm range [60,61]; others indicate that the dependence is not that straightforward [62]. Another important question is whether one uses nanoparticles, nanotubes, or nanosheets. For carbon, it seems that nanotubes are the most toxic, but the use of graphene (nanosheets) is also not without risk [58,59].
3. Clustering of a large number of nano-objects into an aggregate or agglomerate is another important aspect. For example, many commercial nanopowders are produced via flame synthesis [48], while their primary particles are typically 10–30 nm, they sinter during production forming very strong aggregates, typically of several 100s of nm. Due to fact that the particles have formed “necks” between each other, it is nearly impossible that they will detach during their lifetime.
4. Coating or encapsulation is an attractive manner to shield off a potential harmful particle from its environment (e.g., the human body). Scalable approaches to produce precisely coated nanoparticles are available [48]. An example is the coating of TiO_2_ nanoparticles in sunscreen, which maintains the positive property of TiO_2_ (protection against the sun) while averting its drawback (reactive to the skin) [63].
5. Embedding prevents the spreading of nanomaterials by putting them in another material, such as a polymer, where, in principle, they should be contained for their entire lifetime [64]. An example is the use of nanosilica in rubber tires [65].
6. Minimizing quantity is the last aspect. Due to the fact that nanoparticles are typically very active, using a tiny amount is often sufficient. Morose [53] gives an example from the lighting industry, which has significantly reduced the amount of toxic mercury used in fluorescent lights over the years.

#### 4.7.3. Outlook: Organizing Safety Knowledge Base

The special properties of nanomaterials renders them very attractive to use, but because we do not yet know all the aspects involved, their contact with living organisms can be dangerous and should be minimized via design. Therefore, research on nanomaterials’ impacts on health and environment should be increased. A second dilemma is the possible tension with circular product design. There is a trend to move to a society where products are designed for multiple use cycles [66]. However, adding materials in extremely small quantities or strongly embedding them in a product makes it more difficult to reuse that product.

Although clear regulations for “regular” chemicals (REACH) exist, there is no such clarity for nanomaterials [67,68], even though they are already being used in consumer products. More in-depth studies are required to provide more insight into the effect of nanomaterials on the human body and the environment. Ideally speaking, such studies should yield mechanistic insight such that new studies are not needed for every novel material. Such studies could help researchers and developers decide whether a given nanomaterial can be safely applied or whether its design should be adapted accordingly.

### 4.8. Cyberspace 

#### 4.8.1. Context: Security as a Distinct Safety Aspect in Digital Technologies

Software and networking technologies have fundamentally changed societal infrastructures. While society’s dependence on the internet and associated technologies is immense, designers have been struggling to cope with the implications that small mistakes can have regarding malicious activities and data breaches. The question is whether better design methods exist that would improve security and privacy in cyberspace. In this context, researchers have proposed a focus on Security–by-Design [69] and Privacy-by-Design [70].

While safety aims at reducing harm from unintentional events, security aims at reducing harm from intentional events. This means that security engineering [71,72] aims at protection against adversaries (often called attackers), which could be cybercriminals, terrorists, and so on. In addition to harm caused, there is also a gain involved for those adversaries. The model of the threat is therefore typically not only probabilistic but intentional or strategic, incorporating behaviors aimed at achieving those gains. This implies that partial protection is often not enough, because adversaries will search for weak spots to gain the access they require. Regulating access is a fundamental activity in security.

In addition, privacy is often foregrounded as a value to be protected in cyberspace [73]. While security focuses mostly on the threat, privacy focuses mostly on what needs to be protected (the asset) and in this case it is personal data. The key aim of privacy in cyberspace is to give subjects control over their personal data and associated access.

There are several key sources of cybersecurity and privacy problems. First, mistakes made in software programming or communication protocols often provide attackers with opportunities to achieve something that was not intended by the designer. In addition, even when the software is secure, users may be tricked into supplying attackers with essential information that enables them to gain access (social engineering, for example phishing e-mails).

Risk assessment methods play a key role in identifying possible security threats to a system. One has to “think thief” in the sense that one has to be able to imagine what adversaries may be up to. Methods such as misuse cases (as opposed to use cases; Sindre and Opdahl [74]) can help in this respect.

#### 4.8.2. Focus: Accounting for User Needs and Complex Configurations

A key principle in cybersecurity is that the security of a system should not rely on the secrecy of its security mechanisms. The latter approach is often called security-by-obscurity. Transparency of security mechanisms goes back to the so-called Kerckhoffs’s principle [75]. More recently, the OWASP (Open Web Application Security Project) development guide [76] lists additional core security principles for software development.

In addition, developers should take into consideration the effect security designs will have on users. Security typically makes it more difficult to do certain tasks because the system needs to be able to distinguish between legitimate and illegitimate access, for example, through forms of access control (passwords, SMS, etc.). If security becomes too cumbersome, users may find workarounds and, thereby, possibly compromises security [77]. Simply subjecting users to awareness campaigns may not be effective; the design itself needs to incorporate the intended behavioral change in a sociotechnical context. Secure (or privacy-friendly) defaults are a key design pattern here.

A central design choice for privacy is between centralized and decentralized architectures. In a centralized architecture, there is a single point where all data of all users can be accessed, whereas data is only stored locally in a decentralized architecture. Since free services often rely on data as a form of payment (dubbed “surveillance capitalism”), centralized architectures have become the norm in the commercial domain.

Given the complexity of software, it seems almost inevitable that mistakes, which may be security-critical, will be discovered after deployment. Therefore, not all security can be handled at the design stage and it is important to facilitate the discovery and repair of software bugs when the software is already being used. Complementary to security-by-design, this could be called security-by-experiment [78]. Responsible disclosure is an important feature in this type of security. Ethical hackers are invited to submit issues they find and may even receive a reward for their contributions to security.

Due to its focus on adversaries, security has an inherently political dimension: security of whom against whom? For example, should we, as a society, be protecting the privacy of users or the rights of copyright owners on file sharing services? Should we facilitate encryption to ensure secret communication under oppressive regimes or should we limit the use of encryption to enable monitoring communications of suspected terrorists? Security-by-design may not work if we do not agree on what we mean by security or it may run the risk of supporting only one particular interpretation/frame.

Some safety domains (such as structural safety and chemical safety) seem to have widely accepted quantitative metrics, but this is still a big challenge in the security domain (e.g., Sanders [79]) and there is much debate on whether security research can even be a science. This makes it difficult to answer the question of *how much* security should be in a design, especially since more security may limit functionality or usability (which is the case for safety as well).

#### 4.8.3. Outlook: Maintaining Security and Connectivity

A main issue in the future of security-by-design lies in the complexity of the designer network. Code is often reused and this may lead to large-scale vulnerabilities (such as Heartbleed [38]). However, whether increased diverse design (i.e., not using the same software all over the place) would contribute to security is still being debated.

The increased connectivity of physical devices poses another challenge. In the internet of things, many devices are connected and it is often unclear how security is being handled, especially when the devices become older and software updates may no longer be provided. Again, this is not only a design issue but a life cycle one.

Finally, market pressure for software may make it hard for developers to pay sufficient attention to security. Thus, there is a meta-design issue regarding designing an environment for developers that facilitates secure designs (see Ahmed and van den Hoven [80]), for example, by providing the right incentives [81].

## 5. Discussions: Design Methods 

In the previous section, a wide variety of perspectives from engineering disciplines was discussed. This section focuses on the commonalities and more specifically on the design strategies; Table 1 lists these strategies and the associated principles for including safety.

### 5.1. Strategies in Principle and in Practice

The different disciplinary approaches to safety discussed in the previous section reveal a number of risk management strategies at the design level that appears pertinent to understanding what Safe-by-Design entails. We distinguish between different risk-producing technologies: (I) basic technology-oriented risks such as nano, bio, and chemical risks and (II) risks that are more tied to end-users, thereby covering various dimensions. In Table 1, we will first extend and categorize the strategies. Some of these design methods have been explicitly mentioned as a strategy in Section 4. Some other methods as formulated here are the authors’ interpretation of a strategy, while other strategies have not been mentioned as a risk management strategy. The expressions used are the authors’ and they are chosen based on the broad safety science literature. Table 2 extends this approach and investigates whether and to what extent each design method has been used in different engineering disciplines. 

For indicating the visibility of each strategy in the different domains, we have added the number of Google Scholar hits on the respective keywords on the horizontal and vertical axes along with the following domain-specific keywords, using an OR gate:

- “Chemical engineering” OR “process industry”;
- “Nano-engineering” OR nano-technology, nanomaterial, “nanostructured material”, and “nanoengineered material”;
- “Software engineering “ OR “software development”, and “software design”;
- Bio-engineering OR bio-technology;
- “Aerospace engineering” OR aviation;
- “Construction engineering” OR “structural engineering”;
- “Cyber space” OR cyber-physical.

The counts are disturbed by random influences, but they may serve as a rough indicator for the visibility of the design strategy in a given application domain. 

The illustrations in Table 2 underline the many differences in type of risks as well as domain specific responses. In this respect, the very variety of safety issues—as already implied by the list of design strategies in Table 1—shows that safety is not a uniform or unambiguously approached and conceptualized value but a multidimensional challenge. Yet, two related patterns can be identified. First, when comparing disciplines dealing in so-called platform technologies (such as nanotechnology) with disciplines in which applications are more intrinsically part of the research practice (e.g., urban engineering), the intricacy of the entwinement with context is greater in the latter. Second, the more disciplinary knowledge is interwoven with (societal) context, such as in application-oriented disciplines, the more roles are emerging for non-technical expertise in assessing issues of risk and safety. For example, making toxicity assessments that consider the full life cycle of a chemical component obviously requires mostly highly specific types of technical expertise; natural surveillance in the built environment, however, is both something that assumes an active role for ordinary citizens and something that citizens are likely to want to have—and arguably deserve—a say in. 

### 5.2. Context Matters

From bioengineering to software engineering and from aerospace engineering to nanotechnology, what can we meaningfully say about Safe-by-Design by looking at the disciplinary outlooks from such a varied collection? The generic positioning of Safe-by-Design, which views safety as a design requirement from the early stages of product and process development onwards, leaves ample room for interpretation. In addition, Safe-by-Design varies from context to context, depending on what hazards and risks are considered, how safety is conceived of, and what it takes to realize safety through engineering efforts.

To some extent, this variety ties in with the system features of each particular sector. For instance, the fields of nanotechnology and aerospace engineering differ greatly in how they are regulated. Apart from the generic regulation of chemicals, dedicated safety regulations for nanomaterials are being implemented at a very slow rate, while aerospace engineering, which is a firmly established discipline, has multilayered and internationally standardized safety regulations in place. We could also distinguish between these two fields in the types of risk and uncertainty they face. In nanotechnology there is much more uncertainty present than in aerospace engineering with risks for which the natures and probabilities are fairly well known. Given such differences in the sectors in which disciplinary work can feature, it is unsurprising that risk, safety, uncertainty, and Safe-by-Design take on different shapes and significations in these disciplines.

It can be observed that highly institutionalized safety practices, such as in aviation and biotechnology, provide high levels of safety, but they also render it difficult for developers and regulators to adapt to new developments. A field such as nano-engineering, which largely lacks safety regulation specific to the field, requires that the development of technologies, insofar as it is possible, runs synchronous with the development of knowledge of safety concerns and regulatory frameworks for risk assessment and management. This goes to show that strategies developed in one context cannot always be transferred to another. Another case in point: while aviation’s safety culture could in theory be beneficial for dealing with similar safety issues in construction engineering (especially the organizational aspects), in practice, such a culture would be difficult to manage. At the heart of this discrepancy lies the widely divergent structures of both sectors. Whereas the aviation sector counts only a handful of big players, the construction sector comprises a very large number of independently operating actors, each with weaker liability/accountability relations. 

Furthermore, sometimes we find that references are being made to the same or similar concepts in different disciplines but with dissimilar interpretations. For instance, we find that the role of automation in coping with increasing complexity and uncertainty is also different for a number of disciplinary domains. In aerospace, automation is dependent on the ability of authorities to catch up; in process engineering, it serves as quantitative assessment; and in software and construction engineering, it forges safety assessments.

In addition, the closer a discipline is to the end of the so-called innovation pipeline, the more uncertainty is accepted in conceptualizing risks. Specifically, risks tend to be identified in more places, related to more types of (what we have called) problems, and a larger set of potential and partial solutions is considered. This is the case even though addressing risks farther away from the end of the pipeline could potentially be more efficacious and more efficient because the reach of risk management strategies would be far wider.

## 6. Conclusions

The differences and commonalities between safety strategies in different engineering disciplines come with a dual challenge for conceptualizing and operationalizing Safe-by-Design in practice. On the one hand, the accounts presented in Section 4 are all tied in with very specific pathways of historical development. A better understanding of these disciplinary and regulatory histories would contribute to understandings of how the value of safety is practically embodied in different fields. Such context-specific understanding is required for aligning safety practices with other important values at the heart of societal challenges, from climate mitigation to building resilient societies capable of dealing with the pressures of a global pandemic, for instance. There is still much to learn about how to practically pull this off: for example, how to find synergies between values or how to make choices when trade-offs are unavoidable. Given the inherent limitations of anticipation, in order to make steps forward, some forms of learning by doing cannot be avoided. That could for instance mean that researchers, developers, and regulators work together to develop, test, and assess how different safety-oriented design approaches and dedicated governance arrangements for warranting safety and security fare in different contexts and to investigate how best to adapt such approaches in response to both lessons learned and evolved circumstances [83].

On the other hand, this same variety across disciplines and their histories also calls for conceptualizing generic elements in Safe-by-Design approaches. Here, new avenues for research could also follow a more normative lead, not investigating the meanings Safe-by-Design is practically given in different fields and the reasons behind those, but exploring what it would entail if Safe-by-Design were employed as a conceptual yardstick to measure disciplinary practices against. We thus conclude this article by offering a first proposal for such a conceptualization of Safe-by-Design. This conceptualization builds on all the preceding information presented here as well as on considerations regarding the dilemma of control in technological innovations, as discussed in Section 1.

### 6.1. Safe-by-Design as Normative Yardstick: Towards a Value-Inclusive Approach to Innovation 

**Figure 1:**
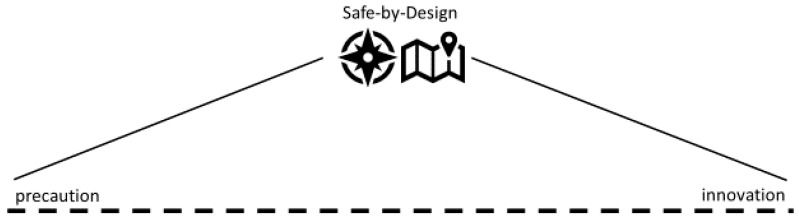
Navigating the Scylla of precaution and the Charybdis of innovation through Safe-by-Design.

We conclude that one of the strengths of Safe-by-Design is that it can help strike a balance between the need for technological innovation that increases substantive values such as well-being, sustainability, or equity on the one hand and the equal need for being more cautious about potential risks that could emerge on the other. The development of new technologies and the control of the unknown risks of those technologies, then, requires a balance between precaution and innovation; a theme that is also topical in the policy and governance of innovation today. While Safe-by-Design clearly resonates with the precautionary perspective, we propose that it can in fact be used to navigate between the typically opposed extremes of precaution and innovation, as illustrated in Figure 1. This couples Safe-by-Design to the mood of learning by performing what we noted before, which builds on the conviction that not knowing everything in advance need not stymie innovation. Before turning to the normative relevance of this conceptualization of Safe-by-Design, we will briefly make the case that this notion constitutes a method for combining the strengths of precaution and innovation, while mitigating their respective weaknesses.

That Safe-by-Design fits with the perspective of precaution is clear: Safe-by-Design is primarily aimed at anticipating risks early in order to enable addressing those risks in the design phase, thereby making materials, products, and technologies safer from the outset. In some disciplines more than in others, the borders of what can be anticipated come into view quite (cl)early, however. Bioengineering and software engineering perspectives, for instance, do not hide the fact that *unknown unknowns* can be expected to be in place where innovation is concerned.

Since addressing safety issues by design entails an innovation-driven approach to solving problems, Safe-by-Design can simultaneously incorporate the innovation perspective. Rather than aiming at stopping or slowing down innovation, Safe-by-Design could help to innovate in socially responsible directions while taking into account the uncertainties associated with these innovations. In that sense, Safe-by-Design may be seen as an operationalization of responsible innovation in that it frontloads safety. To appropriately deal with both the forces of innovation and precaution, we suggest that Safe-by-Design should require innovators and regulators to *also* develop early detection methods for risks, such that it becomes possible to mitigate them once they arrive.

Therefore, Safe-by-Design should aim at creating active monitoring and room for actors in all life phases of products and technologies to address sometimes unexpected safety issues and ultimately aiming at having measures already in place in the design phase that could help prevent or mitigate risk, reduce the likelihood of its occurrence, and limit its potential consequences [7]. In other words, Safe-by-Design is not the privilege of technology developers or designers per se; it is *also* a practical tool for policymakers and risk assessors. It is a tool, then, that helps to shape governance arrangements for accommodating and incentivizing safety, while fully acknowledging uncertainty, and which does so by organizing for responsibility through monitoring practices and facilitating adaptive change.

Safe-by-Design, then, constitutes a form of responsible innovation that simultaneously highlights, reflects, *and* challenges safety as one the leading values in many engineering design perspectives. In some fields—especially those broadly contributing to resolving grand societal challenges—other values are also prominent, including sustainability and circularity (e.g., in chemical engineering and construction engineering), security (e.g., in cyber security), and privacy (e.g., in software engineering). Indeed, we find that in software engineering, for example, there are practices similar to Safe-by-Design that focus on other important values from the outset, for instance, Privacy-by-Design [70]. One can of course design for a plethora of values, *including safety*. This is the focus of the Design for Values field [84]. What, then, is unique about Safe-by-Design? We argue that most (but not all) engineering endeavors should in one manner or another bring about a balance between other values and safety. For example, when pursuing well-being, engineers and designers must ensure that they ward off the risk of undue environmental pressure or, in ensuring safety, they must ensure that they do not jeopardize the value of privacy. Safe-by-Design could thus offer a placeholder to proactively and prominently include safety in design in relation to other important values, such as sustainability or privacy, and with a potentially wider set of stakeholders. In this manner, Safe-by-Design can be used to navigate the extremes of two ideal-typical principles in the field of research and innovation policy: the precautionary principle and the innovation principle. 

### 6.2. Recommendations for Future Research

Pertinent questions for future work in or on Safe-by-Design therefore include questions such as under which conditions can we find the introduction of new technologies acceptable [85] and how safety ought to be weighed against other relevant values. One approach might be adaptive risk governance, which is a form of risk governance that is updated for new experiences and scientific insights [86,87,88]. Thus far, adaptive risk governance has not been coupled to the specific approach to technological innovation Safe-by-Design embodies. Something similar holds for sociopolitical strategies to increase the resilience of sociotechnical systems in which new technologies are implemented [89]. Research and practice in this area has been developed in isolation from the design-centered approach to ensuring safety and realizing values that Safe-by-Design has to offer. It is therefore important to ask whether or how the gaps at the institutional levels and in the levels of abstraction in which risk and safety are discussed within adaptive risk governance and resilient sociotechnical systems can be bridged in order to make contact with Safe-by-Design.

Another important question in pro-actively assessing safety is how we deal with uncertainties; uncertainties could vary in type and different types requires different means for addressing them. Van de Poel and Robaey’s [7] work could serve as a basis for further elaborating a taxonomy of uncertainties pertinent to understanding disciplinary specificities and differences in dealing with safety, along with means for addressing each type of uncertainty.

Finally, future work should focus on spelling out the security aspects in design. As the world is becoming increasingly complex and interconnected, engineering design needs to better accommodate and address safety and security in conjunction; i.e., the complexity opens up new avenues of vulnerability that need to be addressed explicitly. A future Safe and Secure by Design approach could be very helpful for more responsible engineering design, while also shaping up governance practices that could help to appropriately deal with future complex and interconnected risks.

## Figures and Tables

**Table 1:** Design strategies and associated principles.

Design Method		Principle
**A**	Probabilistic risk-based design	Incorporates target reliability indices, system decomposition into subsystems (fault and event trees), and probabilistic models of stress on and capacity of the system in the design.
B	(Deterministic) safety factor-based design	Incorporates multiplication factors on load and resistance variables of the system.
C	Fail-safe design/fail-secure design	In engineering, a fail-safe is a design feature or practice that in the event of a specific type of failure, inherently responds in a way that will cause minimal or no harm to other equipment, to the environment, or to people [82]. Unlike inherent safety to a particular hazard, a system being “fail-safe” does not mean that failure is impossible or improbable, but rather that the system’s design prevents or mitigates unsafe consequences of the system’s failure. That is, if and when a “fail-safe” system fails, it remains at least as safe as it was before the failure.
D	Active safe design	Involves a reaction to a dangerous event by user intervention. For example, in the car industry, active safety measures are already in operation prior to an accident.
E	Passive safe design	Involves a reaction to a dangerous event automatically by natural laws.
F	Vandal-proof design	Design against vandalism.
G	Idiot-proof/fool-proof design	Design against misuse by end-users or to minimize negative consequences of abuse.
H	Fault-tolerant design	System continues processing (possibly at a reduced level) when part of the system fails.
I	Circular design	Design that enables maintaining product integrity (i.e., functionality and value) over a long period of time and eliminates waste.

**Table 2:** Manifestations of risk management strategies in different disciplines.

	**Historically Grown** **Technologies**	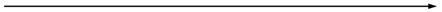						
**Disciplines → Design methods ↓**	**Construction engineering**	*GS hits*	**Chemical engineering**	*GS hits*	**Aerospace engineering**	*GS hits*	**Urban engineering**	*GS hits*
**A** **Probabilistic risk-based design**	Target failure probabilities are specified, depending on the failure consequences of the structures.	2510	Process safety design: Identify failure scenarios and estimate consequences; redesign until risks are below a target level (of 10^−6^/year/individual).	361	Acceptable level of risk calculations based on components.	1820	Related to the probabilistic design of infrastructure within the urban environment.	219
**B** **Safety factor-based design**	Multiplication factors are used on characteristic/ representative values of load and resistance variables.	15,100	Anticipate higher loads and weaker resistance by incorporating safety factors in the design.	4820	Example: Determining maximum load for strength of wings and other structures.	12,700	As above.	2570
**C** **Fail-safe design/ fail-secure design**	Failure of one construction element does not lead to complete collapse.	2070	Replace materials with less-hazardous options (e.g., clean with water and detergent rather than a flammable solvent).	1960	Example: Statically stable design of aircraft, which means failure of automatic flight control system does not lead to an uncontrollable aircraft.	17,600	Related to resilience of the urbanized area.	1280
**D** **Active safe design**	Actively monitor the construction site to prevent accidents and fatalities.	374	Use of sensor and control technology to stabilize pressure and temperature levels.	357	Example: Traffic collision avoidance system warns of traffic and advises pilots.	2360	Focused on active safe design of road infrastructure in the urban environment.	672
**E** **Passive safe design**	Use passive safe columns to absorb the energy of a collision.	333	Gravity taking leaks to safe places; use bunds; avoid knock-on effects.	730	Example: Crash structures and seat design.	1750	Focused on passive safe design of hazardous industries inside urbanized areas.	222
**F** **Security-proof/ vandalism-proof design**	Use gates, fences, or surveillance cameras.	8	Use gates, fences, or surveillance cameras.	23	None: Aircraft operate in secure areas and people inside aircraft generally do not want to put themselves in danger.	177	Crime prevention by improving natural surveillance in the urban environment.	8800
**G** **Idiot-proof/fool-proof design**	Careful supervision of design and execution phases of the construction project.	432	Make incorrect assembly impossible; ease of control.	264	MINIMAL: Airbus aircraft have built in protection against aircraft upset due to incorrect pilot inputs. Highly skilled end-users (pilots) are expected.	4280	None.	0
**H** **Fault-tolerant design**	Space between construction elements to accommodate fluctuations in geometrical dimensions.	1520	Equipment and processes designed to withstand possible faults or deviations from design.	2870	All crucial systems are redundant, sometimes triply or quadruply. A single fault should never lead to a crash.	20,400	Related to infrastructural design of the built environment.	2220
**I** **Circular design**	Modular construction strategies (“Lego-type” structures).	125	Redefine performance to include entire product life cycle; “Nexus” solutions that synergistically solve several sustainability issues.	115	MINIMAL: Aircraft are mainly designed for their operational phase. After the operational phase, aircraft are stored or scrapped.	417	Green cities.	107
**Disciplines → Design methods ↓**	**Software engineering**	*GS hits*	**Bio-engineering**	*GS hits*	**Nano-engineering**	*GS hits*	**Cyber space**	*GS hits*
**A** **Probabilistic risk-based design**	Use probabilistic programming or probablistic verification to take uncertainties into account; (For this column we take the software developer’s perspective not the user of the software).	514	Escape frequencies as a measure.	232	For example, the spread of various areas and species in the environment.	97	Explicit modelling of threat actors and their behavior may provide guidance regarding risk level and associated controls.	98
**B** **Safety factor-based design**	Make software forewards compatible by anticipating on future functional and safety requirements.	3280	Found in rationales of SbD but implementation limited.	5840	Limiting release may be combined with limiting toxicity.	846	Security measures such as cryptographic key lengths should consider future developments (e.g., increased computing power).	390
**C** **Fail-safe design/fail-secure design**	Use software verification or static analysis tools to ensure that certain properties hold by construction.	12,500	Closest to the technical application of Safe-by-Design (e.g., kill switches).	6350	Naomaterials used to make fail-safe (construction) materials; rarely used to make nanomaterials themselves safe.	445	Intrusion prevention systems aimed at reducing damage from a detected cyberattack.	2690
**D** **Active safe design**	Programmer manually writes tests or uses program analysis tools to ensure software quality.	2040	Closest to the technical application of Safe-by-Design (e.g. biosensors).	547	Mostly, nanomaterials used in components for active safety; rarely used to make nanomaterials themselves safe.	77	Network monitoring may indicate attacker activity and enable operator responses.	767
**E** **Passive safe design**	Testing or program analysis tools are integrated in the software development pipeline through continuous integration.	1180	Closest to the technical application of SbD (e.g. auxotrophy).	548	Mostly, nanomaterials used in components for passive safety; rarely used to make nanomaterials themselves safe.	124	Decentralized architectures limit the amount of data accessible through a single system.	253
**F** **Security-proof / vandalism-proof design**	Explicitly validate inputs to provide robust response to all possible inputs, for example, to prevent injection attacks.	1430	None.	3	None.	0	Backups and quick restore procedures make cyber attacks and vandalism less attractive.	317,000
**G** **Idiot-proof / fool-proof design**	Testing or program analysis tools are integrated in the software development pipeline through continuous integration.	3870	Can be understood as biosecurity, not presently covered.	3130	Typically refers to synthesis and applications and not so much to safety.	219	Privacy-friendly or security-friendly defaults (e.g., multi-factor authentication) may protect users against attacker manipulation (e.g., phishing e-mails).	2610
**H** **Fault-tolerant design**	Explicitly validate inputs to provide robust response to all possible inputs, for example, overflows and illegal memory access.	17,500	None. Not in the rationales of SbD.	4190	No relation to toxicity.	0	Network segmentation limits possibilities for attackers to compromise the whole system.	12,000
**I** **Circular design**	Reuse of software through libraries, thereby inheriting safety guarantees of the libraries.	303	None. Is found in bioengineering but not discussed in the context of safety.	371	Possible tension between Safe-by-Design and circular design (see text).	81	Adequate identification of and response to software vulnerabilities, via software updates, is crucial.	78

Note: GS = Google Scholar.

## Data Availability

The data presented in this study are available directly from the current article in Table 2.
